# Decomposition Analysis of Wastewater Pollutant Discharges in Industrial Sectors of China (2001-2009) Using the LMDI I Metho

**DOI:** 10.3390/ijerph9062226

**Published:** 2012-06-14

**Authors:** Hongjun Lei, Xunfeng Xia, Changjia Li, Beidou Xi

**Affiliations:** 1 State Key Laboratory of Environmental Criteria and Risk Assessment, Chinese Research Academy of Environmental Sciences, No. 8 Dayangfang Road, Chaoyang District, Beijing 100012, China; Email: hj_lei2002@163.com (H.L.); xiaxunfengg@sina.com (X.X.); 2 School of Water Conservancy, North China University of Water Conservancy and Hydroelectric Power, No. 36 Beihuan Road, Zhengzhou, Henan 450011, China; Email: lcjyzh@gmail.com

**Keywords:** decomposition analysis, industrial wastewater pollutant discharges, LMDI I method, China

## Abstract

China’s industry accounts for 46.8% of the national Gross Domestic Product (GDP) and plays an important strategic role in its economic growth. On the other hand, industrial wastewater is also the major source of water pollution. In order to examine the relationship between the underlying driving forces and various environmental indicators, values of two critical industrial wastewater pollutant discharge parameters (Chemical Oxygen Demand (COD) and ammonia nitrogen (NH_4_-N)), between 2001 and 2009, were decomposed into three factors: *i.e.*, production effects (caused by change in the scale of economic activity), structure effects (caused by change in economic structure) and intensity effects (caused by change in technological level of each sector), using additive version of the Logarithmic Mean Divisia Index (LMDI I) decomposition method. Results showed that: (1) the average annual effect of COD discharges in China was −2.99%, whereas the production effect, the structure effect, and the intensity effect were 14.64%, −1.39%, and −16.24%, respectively. Similarly, the average effect of NH_4_-N discharges was −4.03%, while the production effect, the structure effect, and the intensity effect were 16.18%, −2.88%, and −17.33%, respectively; (2) the production effect was the major factor responsible for the increase in COD and NH_4_-N discharges, accounting for 45% and 44% of the total contribution, respectively; (3) the intensity effect, which accounted for 50% and 48% of the total contribution, respectively, exerted a dominant decremental effect on COD and NH_4_-N discharges; intensity effect was further decomposed into cleaner production effect and pollution abatement effect with the cleaner production effect accounting for 60% and 55% of the reduction of COD and NH_4_-N, respectively; (4) the major contributors to incremental COD and NH_4_-N discharges were divided among industrial sub-sectors and the top contributors were identified. Potential restructuring and regulation measures were proposed for pollutant reduction.

## 1. Introduction

**Figure 1:**
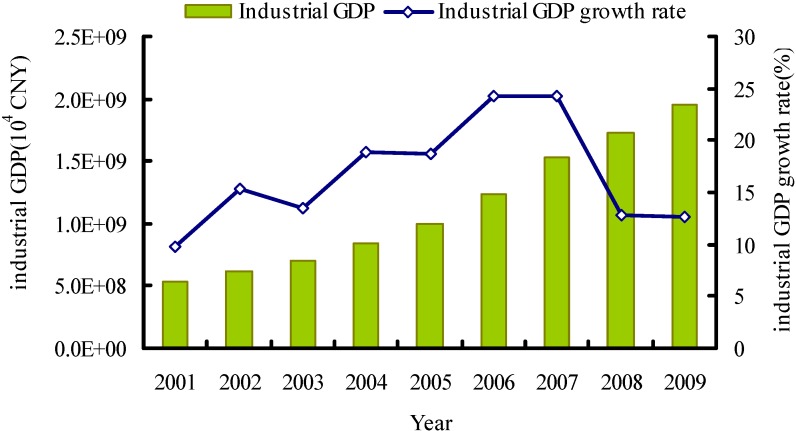
Changes of China’s industrial gross domestic product during 2001–2009 (Data resources: Annual Statistic Report on Environment in China—ASREC [4]).

If any region can be seen as a microcosm with both the environmental and developmental problems and opportunities facing the World, particularly the developing countries, it is China. Since 1978 China’s gross domestic product (GDP) has experienced an annual growth of ten percent, making China the fastest growing country [1]. China’s industrial GDP showed remarkable growth rate of over 10% between 2001 and 2009 (see Figure 1). In 2007, China’s GDP stood at US$3.38 trillion, which made China the world’s third largest economy as far as GDP is concerned [2]. China’s economic growth, industrialization, and urbanization coupled with inadequate investment in basic water supply and treatment infrastructure have resulted in widespread water pollution, and China is quickly moving in the direction of becoming one of the world’s biggest polluters. A report from United Nations Environmental Programme (UNEP) demonstrated that environmental damages were resulting in significant losses of GDP in China each year [3]. More and more attention has been paid to whether or not the country’s development is sustainable and when and how its environmental conditions will be improved.

In 1991, Grossman innovatively put forward the concept of Environmental Kuznets Curves (EKC) to explain the relationship between economic development and environmental quality. The process can be expressed as an inverted “U”-shape *i.e.*, three stages from harmony to disorder, followed by coordination [5]. However, the inverted “U”-shape is not the sole possible relationship between the two, so economic development is not the exclusive solution to environmental problems [6,7]. To identify the underlying mechanisms of economic progress and environmental degradation, several theories and empirical methods were put forward during the past three decades, *i.e.*, the econometric regression, the structural decomposition analysis (SDA) and the index decomposition analysis (IDA) [8]. IDA is a useful technique for quantifying a large number of underlying factors that contribute to the changes in environmental quality by decomposing these changes at the sector level. The advantage of IDA is that it can be readily applied to various available data at different levels of aggregation [9]. With respect to IDA, there have been a variety of different index methods available, such as Laspeyres index [10] and the Divisia index [11,12]. In 1997, Ang and Choi [13] first put forward the multiplication version of the Logarithmic Mean Divisia Index (namely LMDI II), which resolved the residual problem in the Divisia index method but led to a new problem of discontinuity in aggregation. Ang and Liu proposed the additive version of the Logarithmic Mean Divisia Index (LMDI I), which was complete in decomposition and consistent in aggregation, and efficient in handling zero values [14]. LMDI I is widely used in the industrial energy conservation, and SO_2_ & CO_2_ emission reduction analysis [15,16]. However, few attempts have focused on the interaction analysis between the industry wastewater pollutant discharges and the economic growth.

China’s 12th Five-Year Plan (2011–2015) aims to achieve an average annual GDP growth of 7% while protecting the environment with an aim to reduce emissions of chemical-oxygen demand (COD) and ammonia nitrogen (NH_4_-N) by 8% and 10%, respectively, over the five-year period. In order to achieve harmonized development between economic growth and environmental sustainability, China must strengthen its environmental regulations.

**Figure 2:**
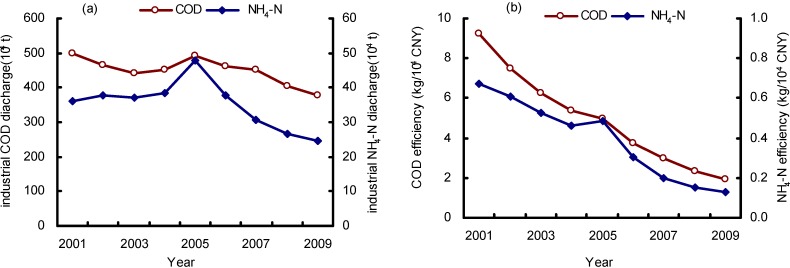
Changes of the wastewater discharges in industrial wastewater during 2001–2009, (**a**) pollutant discharges; (**b**) pollutant discharge efficiency (Data resources: ASREC [4]).

During the period of 2001–2009, industrial COD and NH_4_-N discharges in wastewater showed an overall declining tendency (see Figure 2a)and the average annual decreases were 148,927.7 t and 14,580.3 t, respectively. The discharge efficiency (amount of pollutant discharges per 10^4^ CNY, in Figure 2b) of COD and NH_4_-N shows an obvious decline trend from the year 2001 to the year 2009 with the exception of 2005. It is important to know the cause of this change, and to achieve a clear understanding of the relationships among the economic development, the technology improvement, the structure adjustment and the wastewater pollutant discharges. This will make contribution for the policy and regulations making to ensure the realization of COD and NH_4_-N reduction goal.

This paper analyzes the impact of different factors on the change of China’s industrial COD and NH_4_-N discharges. The aim of the paper is to identify the dominant factors driving the change of these COD and NH_4_-N discharges. Based on the two critical pollutants emission from industrial wastewater, the representative IDA method LMDI I is used to demonstrate the interaction mechanism. The changes in industrial pollutant discharges were decomposed into the scale of economic activities (production effect), the technological level of each sector (intensity effect), and the economic structure (structure effect).

This paper is organized as follows: Section 2 describes the LMDI I method and the data used. Section 3 presents the impacts of different factors on the changes of China’s industrial COD and NH_4_-N discharges based on LMDI I. Section 4 presents the conclusions, and the last section offers some policy implications.

## 2. Methodology and Data

### 2.1. Principle and Basic Model

IDA is a useful and meaningful tool for quantifying a large number of underlying driving forces that contribute to changes. The underlying theory of IDA is the definition of a governing function relating an aggregate indicator to a number of pre-defined factors of interest, thus identifying the respective effects of the factors on the aggregate [17]. According to Sun [18], changes in China’s industrial wastewater discharges can be attributed to production effects, intensity effects, and structure effects. In addition, the intensity effect can be divided into cleaner production effect and pollution abatement effect. To illustrate how the IDA approaches work, some related variables are listed in Table 1.

**Table 1:** Summary of notations and definitions.

Variables	Meaning of variables
*E_i,t_*, *E_i,0_*	Industrial pollutant discharge in sector *i* in year *t* and year 0, respectively
*E_t_*, *E_0_*	Total industrial pollutant discharge in year *t*(*E_t_*= ∑*_i_ E_i,t_*) and year 0, respectively
*e_i,t_*, *e_i,0_*	Industrial pollutant production in sector *i* in year *t* and year 0, respectively
*e_t_*, *e_0_*	Total industrial pollutant productionin year *t*(*e_t_*= ∑*_i_ e_i,t_*) and year 0, respectively
*G_i,t_*, *G_i,0_*	Production of industrial sector *i* in year *t* and year 0, respectively
*G_t_*, *G_0_*	Total industrial production in year *t*(*G_t_*= ∑*_i_ G_i,t_*) and year 0, respectively
*S_i,t_*, *S_i,0_*	Production share of industrial sector *i* in year *t*(*S_i,t_*= *G_i,t_*/*G_t_*) and year 0, respectively
*I_i,t_*, *I_i,0_*	Discharge intensity in sector *i* in year *t*(*I_i,t_* = *E_i,t_*/*G_i,t_*) and year 0, respectively
*T_i,t_*, *T_i,0_*	Production intensity in sector *i* in year *t*(*T_i,t_*= *e_i,t_*/*G_i,t_*) and year 0, respectively
*A_i,t_*, *A_i,0_*	Discharge rate in sector *i* in year *t*(*A_i,t_*= *E_i,t_*/*e_i,t_*) and year 0, respectively
Δ *E_tot_*	Total change in industrial pollutant discharge from year 0 to *t*(Δ*E_tot_*= *E_t_* −*E_0_*)
Δ *E_pdn_*	Impact of production change on the aggregate industrial pollutant discharge
Δ *E_str_*	Impact of structural change on the aggregate industrial pollutant discharge
Δ *E_ity_*	Impact of intensity change on the aggregate industrial pollutant discharge
Δ *E_tec_*	Impact of clean technologies change on the aggregate industrial pollutant discharge
Δ *E_aba_*	Impact of pollution abatement change on the aggregate industrial pollutant discharge
Δ *E_rsd_*	Residual terms

Grossman and Krueger [19] and De Bruyn [20] adopted the basic IDA model in the environmental quality analysis. In this paper, we express the aggregate industrial wastewater pollutant discharges as a summation of all sectors, and decompose the impact index of industrial wastewater pollutant discharges as follows:


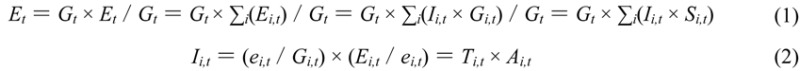


### 2.2. Methodology

Decomposition can be carried out either multiplicatively or additively with the same data of *i* industrial sectors. The additive case is the direct decomposition of a quantity change, which is provided in the chosen measurement unit as the factorized effects. The multiplicative case measures the change by dividing the aggregate intensity of one year, with the factorized effects provided in indices [21]. In summary, different decomposition methods involve approaches formulated differently, which lead to different estimates on the right hand side of the equations. Among the two methods, the results of additive decomposition can be easily to understand and “difference” change is decomposed, whereas the results of multiplicative decomposition can be graphically presented and the “ratio” of an aggregate is obtained.

In additive decomposition, the total change (Δ*E_tot_*) is decomposed into three different effects associated with the following factors: Δ*E_pdn_* (production effect), Δ*E_str_* (structure effect), and Δ*E_ity_* (intensity effect). Moreover, the intensity effect (Δ*E_ity_*) is decomposed into two different effects, *i.e.*, Δ*E_tec_* (clean-production effect), and Δ*E_aba_* (pollution abatement effect). The additive decomposition takes the form below:


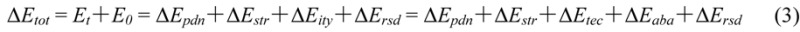


If Δ*E_rsd_* = 0, the method is considered as a perfect decomposition with no residual terms. Here, the additive *LMDI I* is presented below:


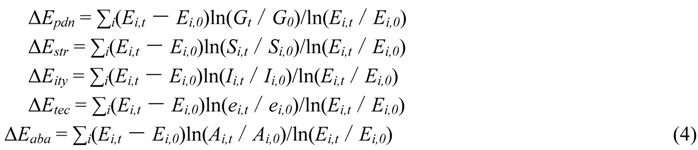


### 2.3. Data Source

Since 2001, China’s industrial sectors have been divided into 40 sub-sectors (see appendix Table A1) in the China Statistical Yearbook (CSY) [22]. Considering the data availability, consistency and the detailed classification of industrial sub-sectors, the time-series data from 2001 to 2009 issues of ASREC [4] and CSY [22] were obtained. In this study, industrial added values at current prices are converted into 2001 constant prices. Since the price indices are not available at the levels of industrial sub-sectors, the added values at constant prices for each sector are derived using the price indices of the industrial sector.

## 3. Results and Discussion

Based on LMDI I, the influencing factors of China’s industrial COD and NH_4_-N discharges in wastewater were analyzed between 2001 and 2009. A positive value of contribution indicates an incremental effect on industrial pollutant discharges, while a negative value indicates a decremental effect.

### 3.1. Yearly Comparisons

The yearly contribution of different effects to China’s industrial COD and NH_4_-N discharge were given in Table 2 and Table 3. The average change of industrial NH_4_-N discharges decreased year by year and the results were given in Table 2. As shown in Table 2, from 2001 to 2009 the industrial COD discharges decreased on average 14.89 × 10^4^ t, and the effects brought about by the production scale, structural scale, intensity scale, cleaner production scale and pollution abatement scale were 72.97 × 10^4^ t, −6.93 × 10^4^ t, −80.94 × 10^4^ t, −53.37 × 10^4^ t and −27.57 × 10^4^ t, respectively. Similarly, the average change of industrial NH_4_-N discharges was −1.46 × 10^4^ t, and the Δ*E_pdn_*_,_, Δ*E_str_*_,_, Δ*E_ity_*_,_, Δ*E_tec_*_, _and Δ*E_aba_* were 5.85 × 10^4^ t, −1.04 × 10^4^ t, −6.27 × 10^4^ t, −3.43 × 10^4^ t and −2.84 × 10^4^ t, respectively. Among the change effect of the different influencing factors, the average value of intensity scale ranked first and the value was mostly negative, indicating that intensity scale was the most effective measures to control pollutants emission, which were confirmed by the study of SO_2_ emissions in China [12] and SO_2_ emissions in Germany and Holland [20].

Table 3 suggests that the average annual change effect of COD discharges was −2.99%, and the effects brought by production scale, structural scale, intensity scale, cleaner production and pollution abatement were 14.64%, −1.39%, −16.24%, −10.71% and −5.53%, respectively. Likewise, the average effect changes of NH_4_-N discharges was −4.03%, and the Δ*E_pdn_*_,_, Δ*E_str_*_,_, Δ*E_ity_*_,_, Δ*E_tec_*_, _and Δ*E_aba_* were 16.18%, −2.88%, −17.33%, −9.48% and −7.85%, respectively.

#### 3.1.1. Production Effect

Between 2001 and 2009, China’s industrial sectors experienced rapid growth. As a result, the production effects (Δ*E_pdn_*) of China’s industrial pollutants (including COD and NH_4_-N) discharges were always positive with COD ranging from 46.40 × 10^4^ t to 103.29 × 10^4^ t and NH_4_-N ranging from 3.03 × 10^4^ t to 9.16 × 10^4^ t (shown in Table 2).

**Table 2:** Contribution of different effects to China’s industrial COD and NH_4_-N discharges (Unit: 10^4^ t).

Period	COD						NH_4_-N					
	Δ *E_pdn_*	Δ *E_str_*	Δ *E_ity_*	Δ *E_tec_*	Δ *E_aba_*	Δ *E_tot_*	Δ *E_pdn_*	Δ *E_str_*	Δ *E_ity_*	Δ *E_tec_*	Δ *E_aba_*	Δ *E_tot_*
2001–2002	68.17	−16.27	−86.53	74.03	−160.56	−34.63	5.23	−3.58	0.02	2.87	−2.84	1.68
2002–2003	57.22	14.69	−93.93	−76.66	−17.28	−22.03	4.73	3.61	−9.09	−8.95	−0.15	−0.76
2003–2004	77.13	−17.49	−49.63	−54.14	4.51	10.01	6.52	−3.56	−1.48	1.78	−3.26	1.47
2004–2005	80.63	−15.83	−23.29	−47.14	23.84	41.51	7.33	−1.12	3.25	0.63	2.62	9.46
2005–2006	103.29	9.08	−142.98	−126.66	−16.32	−30.61	9.16	0.51	−20.11	−11.90	−8.21	−10.43
2006–2007	99.14	17.78	−126.41	−121.65	−4.76	−9.49	7.35	1.82	−16.13	−13.00	−3.13	−6.96
2007–2008	51.82	−17.06	−82.98	−50.68	−32.30	−48.22	3.46	−4.41	−2.92	2.68	−5.60	−3.86
2008–2009	46.40	−30.31	−41.78	−24.10	−17.68	−25.68	3.03	−1.60	−3.69	−1.56	−2.13	−2.26
Average	72.97	−6.93	−80.94	−53.37	−27.57	−14.89	5.85	−1.04	−6.27	−3.43	−2.84	−1.46

**Table 3:** Contribution of different effects to China’s industrial COD and NH_4_-N discharges (%).

Period	COD						NH_4_-N					
	Δ *E_pdn_*	Δ *E_str_*	Δ *E_ity_*	Δ *E_tec_*	Δ *E_aba_*	Δ *E_tot_*	Δ *E_pdn_*	Δ *E_str_*	Δ *E_ity_*	Δ *E_tec_*	Δ *E_aba_*	Δ *E_tot_*
2001–2002	13.68	−3.26	−17.36	14.86	−32.22	−6.95	14.47	−9.89	0.07	7.93	−7.86	4.65
2002–2003	11.48	2.95	−18.85	−15.38	−3.47	−4.42	13.07	9.98	−25.15	−24.73	−0.41	−2.09
2003–2004	15.48	−3.51	−9.96	−10.86	0.91	2.01	18.04	−9.86	−4.10	4.92	−9.02	4.08
2004–2005	16.18	−3.18	−4.67	−9.46	4.79	8.33	20.26	−3.09	8.98	1.74	7.24	26.14
2005–2006	20.73	1.82	−28.69	−25.42	−3.28	−6.14	25.34	1.41	−55.59	−32.89	−22.70	−28.85
2006–2007	19.90	3.57	−25.37	−24.41	−0.96	−1.90	20.33	5.03	−44.61	−35.94	−8.67	−19.24
2007–2008	10.40	−3.42	−16.65	−10.17	−6.48	−9.68	9.57	−12.20	−8.06	7.42	−15.48	−10.68
2008–2009	9.31	−6.08	−8.38	−4.84	−3.55	−5.15	8.36	−4.42	−10.19	−4.31	−5.88	−6.25
Average	14.64	−1.39	−16.24	−10.71	−5.53	−2.99	16.18	−2.88	−17.33	−9.48	−7.85	−4.03

The average production effects on the changes of COD and NH_4_-N discharges were 72.97 × 10^4^ t and 5.85 × 10^4^ t, respectively, indicating the expansion in economic scale has caused an increase in water pollutant discharges. Year 2001–2006 witnessed a rapid increase in economic scale, and the production effect showed a rising tendency with an average annual value of 12.84% (see Table 3). This may be caused by the implementation of macroeconomic policies like expansion of domestic demand and increase of investment, which encouraged a large number of infrastructure construction and industrial projects. Industrial GDP growth rate remained at an average value of 18.11% (see Figure 1), and it made the economic development a major contributor to industrial COD and NH_4_-N discharges. From 2007 to 2009, the industrial GDP kept declining, finally down to 12.59% in 2009, and the corresponding production effect also fell to a certain extent. This is probably because in the Seventeenth National Congress of the Communist Party of China in 2007, China promoted the conservation culture by basically forming an energy- and resource-efficient and environment-friendly structure of industries, pattern of growth and mode of consumption. It is reasonable to conclude that the continued growth in industrial GDP scale accelerated COD and NH_4_-N discharges. The slower the GDP grows the smaller the contribution it makes to COD and NH_4_-N discharges. Therefore, China will still have to face the pressure from GDP growth and industrial pollutions discharges in the foreseeable future.

#### 3.1.2. Structure Effect

Unlike the production effect (Δ*E_pdn_*), the structure effect (Δ*E_str_*) showed no significant regularity. The structure effect was relatively stable, as shown in Table 2 and Table 3. The change values of COD was ranging from −30.31 × 10^4^ t (−6.08%) to 17.78 × 10^4^ t (3.57%), while in the majority of cases it was negative and the average annual value was −6.93 × 10^4^ t (−1.39%); while in the case of NH_4_-N, the change values were between −4.41 × 10^4^ t (−12.20%) and 3.61 × 10^4^ t (9.98%), whereas in most instances it was negative and the average annual value was −1.04 × 10^4^ t (−2.88%). Consequently, changes of economic structure in China’s industry played a certain role in reducing COD and NH_4_-N discharges, but to a small extent. Through the analysis of atmospheric pollutants emissions in the American Midwest during the period of 1970–2000, Tao *et al.* also concluded that the decrease of emission level could be contributed to by 80% by the intensity effect and 20% by the structure effect [23]. The reason may lie in that the industrial structural change over the past nine years is insufficient because of slow change effect. Therefore, industrial water pollutant reduction by structure adjustment should be a long-term policy goal. In respect of industrial pollutants reduction, China’s economic structure optimization has a long way to go.

#### 3.1.3. Intensity Effect

As shown in Table 2, in most cases the intensity effect (Δ*E_ity_*) showed the maximum negative values among the influencing factors for both COD and NH_4_-N, indicating that the industrial pollutants reduction was mainly due to the technological progress in the industrial sectors. Particularly, the average total change (Δ*E_tot_*) of industrial COD discharges during 2001 and 2009 was −14.89 × 10^4^ t, while the change brought by production effect, intensity effect and structure effects were 72.97 × 10^4^ t, −6.93 × 10^4^ t and −80.94 × 10^4^ t, respectively. Obviously, it was the intensity effect that accounted for 543% of the average total change of industrial COD discharge that pushed industrial COD discharges downward. It was the same situation in NH_4_-N discharges. The intensity effect is the overall reflection of various environment laws and regulations, tax policies, and other measures, taking direct investment in pollution control as an example. A dominant intensity effect manifests the effectiveness of the various government measures used to reduce pollutant discharges.

As shown in Table 2, the average intensity effect on the changes of COD discharges was −80.94 × 10^4^ t, the cleaner production effect (Δ*E_tec_*) and pollution abatement effect (Δ*E_aba_*) were −53.37 × 10^4^ t and −27.57 × 10^4^ t, respectively, indicating that both the cleaner production effect and the pollution abatement effect had a decremental effect on industrial COD discharges. Furthermore, the cleaner production effect and pollution abatement effect accounted for 66% and 34% of the intensity effect, respectively, while in the case of NH_4_-N the ratios were 55% and 45%, respectively, indicating that the cleaner production effect played a major role.

**Figure 3:**
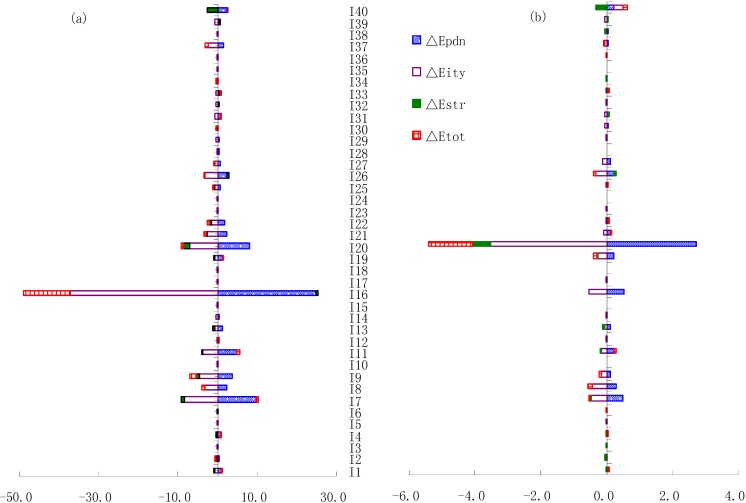
The average changes of industrial pollutant discharges for all sub-sectors between 2001 and 2009 (**a**) COD; (**b**) NH_4_-N.

### 3.2. Sector Comparisons

As the industrial wastewater pollutant discharges are the sum of all sub-sectors, it is essential to decompose the contribution of each sector to the production effect, structure effect, intensity effect, cleaner production effect and pollution abatement effect. The results are given below.

#### 3.2.1. COD Case

Figure 3a showed that there was an increase in COD discharge from fifteen industrial sectors (including textile manufacture, processing of food from agricultural products, mining and processing of non-ferrous metal ores and so on), and the total increment was 3.17 × 10^4^ t, accounting for −21.32% of the average total change; the discharges of twenty-five industrial sectors (including manufacture of paper and paper products, manufacture of beverages, manufacture of foods and so on) decreased, and the total decrease was −18.07 × 10^4^ t, accounting for 121.32% of the total change.

The main contributors to the production effect of COD emissions were manufacture of paper and paper products, processing of food from agricultural products, manufacture of raw chemical materials and chemical products, manufacture of textiles and manufacture of beverages (see Table 4). These five sectors increased by 69.63% the total production effect over the period of 2001–2009. Therefore, these five sectors should be the top sectors to reduce industrial COD emissions. One potential option to reduce industrial COD emission, or at least limit its growth rate would be policies to slow down the expansion of these sectors’ economic scale or encourage restructuring to less COD discharge sectors.

**Table 4:** The top five contributing sub-sectors to the average production effect of industrial COD discharges (10^4^ t).

Industry sectors	Production effect
Manufacture of paper and paper products	27.74
Processing of food from agricultural products	9.72
Manufacture of raw chemical materials and chemical products	7.96
Manufacture of textile	4.78
Manufacture of beverages	3.60
Subtotal of the five sub-sectors	50.81
Total sum of all industrial sub-sectors	72.97

The top six contributors to the accumulated structure effect increased 2.361 × 10^4^ t due to an increase in output shares (see Table 5). However, over the same period there was a decrease in industrial COD emission, thanks to other sectors like manufacture of raw chemical materials and chemical products and processing of food from agricultural products (see Figure 3a).

**Table 5:** The top six contributing sub-sectors related to the structure effectand share rate of COD changes between 2001 and 2009.

Industry sectors	Share rate (%)		ΔEstr (10^4^ t)
	2001	2009	
Manufacture of paper and paper products	2.25	2.83	0.693
Smelting and pressing of ferrous metals	7.78	10.22	0.516
Production and distribution of water	0.10	0.05	0.430
Manufacture of communication equipment, computers and Other electronic equipment	4.04	11.35	0.313
Manufacture of electrical machinery and equipment	0.51	2.27	0.234
Manufacture of transport equipment	7.26	10.46	0.175

The total amount of COD decrement in the structure effects in these three sectors was 5.30 × 10^4^ t, and this offset accounted for 224.36% of the average total structure effects of the top six sectors (presented in Table 5) between 2001 and 2009. Furthermore, the clean-technology effect of other sectors accounted for 84% of the intensity effect and had an incremental effect on COD discharge, and this might indicate that the other sectors include some sectors with low clean-technology; while the pollution abatement effect for mining of other ores played a major role in the increase of COD discharges. So these two industrial sectors should be the top sectors to boost their intensity effect.

#### 3.2.2. NH_4_-N Case

Overall, during the period of 2001–2009, the average total change of China’s industrial NH_4_-N discharge in wastewater was −1.46 × 10^4^ t, and the effects brought by economic scale, economic structure and technological level of the sector were 5.85 × 10^4^ t, −1.04 × 10^4^ t, and −6.27 × 10^4^ t, respectively. Figure 3b showed that there was an increase in NH_4_-N discharge from twenty-two industrial sectors (including mining and washing of coal, other sectors, manufacture of textile and so on), and the total increment was 0.39 × 10^4^ t, accounting for −26.74% of the average total change; the discharges of eighteen industrial sectors (including manufacture of general purpose machinery, mining and processing of nonmetal ores, mining of other ores and so on) decreased, and the total decrement was −1.85 × 10^4^ t, accounting for 126.74% of the average total change. The main contributors to the average production effect of industrial NH_4_-N discharges were manufacture of raw chemical materials and chemical products, manufacture of paper and paper products, processing of food from agricultural products, manufacture of foods, manufacture of textile and smelting and pressing of ferrous metals (see Table 6). These six sub-sectors increased 76.08% of the average total production effect over the period of 2001–2009. Therefore, these six sectors should be the top sectors to reduce industrial NH_4_-N discharge.

**Table 6:** The top six contributing sub-sectors related to the average production effect of industrial NH_4_-N discharges (10^4^ t).

Industry sectors	Production effect
Manufacture of raw chemical materials and chemical products	2.72
Manufacture of paper and paper products	0.52
Processing of food from agricultural products	0.48
Manufacture of foods	0.27
Manufacture of textile	0.23
Smelting and pressing of ferrous metals	0.23
Subtotal of the five sub-sectors	4.45
Total sum of all industrial sub-sectors	5.85

The top six contributors to the accumulated structure effect increased 0.117 × 10^4^ t/year due to an increase in output shares (see Table 7). Over the same period, the changes of NH_4_-N emission in output shares for manufacture of raw chemical materials and chemical products and other sectors decreased (see Figure 3b). The total amount of decrement of NH_4_-N emission from the structure effects in these two sectors was 0.87 × 10^4^ t, and this offsets accounted for 745.38% of the average total structure effects of the top six sectors during 2001 and 2009.

**Table 7:** The top six contributing sub-sectors with related to the structure effect and share rate of NH_4_-N changes during 2001–2009.

Industry sectors	Share rate (%)		∆Estr(10^4^ t)
	2001	2009	
Smelting and pressing of ferrous metals	7.78	10.09	0.053
Manufacture of communication equipment, computers and other electronic equipment	4.04	13.33	0.021
Production and distribution of water	0.10	0.60	0.017
Manufacture of transport equipment	7.26	11.42	0.016
Manufacture of electrical machinery and equipment	0.51	3.52	0.006
Manufacture of paper and paper products	2.25	2.21	0.004

Particularly, the effect brought by technological level of other sectors, mining and washing of coal was positive, with the value of 0.262 × 10^4^ t and 0.014 × 10^4^ t, respectively; while the structure effect were −0.339 × 10^4^ t and −0.005 × 10^4^ t, respectively. Thus, the intensity effect of the other sectors, mining and washing of coal didn’t play its due role and the technological level should be improved. Additionally, the effect brought by technological level of other sectors, mining and washing of coal was positive with the cleaner production effect accounting for 98% of the intensity effect.

## 4. Conclusions

Over period of 2001–2009, China’s industrial COD discharges decreased 14.89 million tons, and the contribution by production scale, structural scale, intensity scale, cleaner production scale and pollution abatement scale were 72.97 × 10^4^ t, −6.93 × 10^4^ t, −80.94 × 10^4^ t, −53.37 × 10^4^ t and −27.57 × 10^4^ t, respectively. Likewise, the average changes of industrial NH_4_-N discharges were −1.46 × 10^4^ t, and the Δ*E_pdn_*_,_, Δ*E_str_*_,_, Δ*E_ity_*_,_, Δ*E_tec_*_, _and Δ*E_aba_* were 5.85 × 10^4^ t, −1.04 × 10^4^ t, −6.27 × 10^4^ t, −3.43 × 10^4^ t and −2.84 × 10^4^ t, respectively. The results also indicate that:

(1) Production effect was the major contributor to the increment of COD and NH_4_-N discharges in industrial wastewater, while the intensity effect played a crucial role in COD and NH_4_-N reduction.
(2) As one of the two parts in the intensity effect, the clean-technology effect played a major role in pollution reduction.
(3) Compared with the production effect and intensity effect, the structure effect played a minor role. However, it did decrease industrial pollution discharges between 2001 and 2009, so industrial structure adjustment should be a long-term policy goal in pollution reduction.
(4) The main contributors to the average production effect of the industrial COD discharges were manufacture of paper and paper products, processing of food from agricultural products, manufacture of raw chemical materials and chemical products, manufacture of textile and manufacture of beverages, while the main contributing sub-sectors to the average production effect of the industrial NH_4_-N discharges were manufacture of raw chemical materials and chemical products, manufacture of paper and paper products, processing of food from agricultural products, manufacture of foods, manufacture of textile and smelting and pressing of ferrous metals. Therefore, these sectors should be the top sectors to offsetthe growth of industrial COD or NH_4_-N discharges.

## 5. Policy Implications

China’s 12th Five-Year Plan (2011–2015) is aiming for average annual GDP growth of 7% and environment protection by cutting the release of COD and ammonia nitrogen (NH_4_-N) by 8% and 10%, respectively. In order to achieve harmonized development of both economic growth and environmental sustainability, China must strengthen its environmental regulations. Our research results suggest the following pollution reduction strategies:

(1) Manufacture of paper and paper products, processing of food from agricultural products, manufacture of raw chemical materials and chemical products, manufacture of textile and manufacture of beverages should be the top sectors for industrial COD reduction. Manufacture of raw chemical materials and chemical products, manufacture of paper and paper products, processing of food from agricultural products, manufacture of foods, manufacture of textile and smelting and pressing of ferrous metals should be the top sectors for industrial NH_4_-N reduction. One potential option to reduce industrial COD and NH_4_-N discharged in wastewater, or at least to limit its growth rate would be policies that slow down the expansion of these sectors’ economic scaleor encourage their restructuring and adjustment to less pollution sectors. Policies should be implemented to encourage the growth of low pollution sectors, and to limit the expansion of pollutants-intensive sectors through the reductionof their exports or the expansion of their imports.
(2) Mining of other ores and mining and washing of coal should be the top sectorstoimportor upgrade advanced technologies.
(3) Economic growth should be integrated with harmonized industrial development and industrial pollutants (both COD and NH_4_-N) reduction.

## Appendix

**Table A1:** Industrial sectors and labels.

Industrial sectors	ID		Industrial sectors	ID
Mining and Washing of Coal	I1		Manufacture of Medicines	I21
Extraction of Petroleum and Natural Gas	I2		Manufacture of Chemical Fibers	I22
Mining and Processing of Ferrous Metal Ores	I3		Manufacture of Rubber	I23
Mining and Processing of Non-ferrous Metal Ores	I4		Manufacture of Plastics	I24
Mining and Processing of Nonmetal Ores	I5		Manufacture of Non-metallic Mineral Products	I25
Mining of Other Ores	I6		Smelting and Pressing of Ferrous Metals	I26
Processing of Food from Agricultural Products	I7		Smelting and Pressing of Non-Ferrous Metals	I27
Manufacture of Foods	I8		Manufacture of Metal Products	I28
Manufacture of Beverages	I9		Manufacture of General Purpose Machinery	I29
Manufacture of Tobacco	I10		Manufacture of Special Purpose Machinery	I30
Manufacture of Textile	I11		Manufacture of Transport Equipment	I31
Manufacture of Texile Wearing Apparel, Footware, and Caps	I12		Manufacture of Electrical Machinery and Equipment	I32
Manufacture of Leather, Fur, Feather and Related Products	I13		Manufacture of Communication Equipment, Computers and Other Electronic Equipment	I33
Processing of Timber, Manufacture of Wood, Bamboo, Rattan, Plam, and Straw Products	I14		Manufacture of Measuring, Instruments and Machinery for Cultural Activity and Office Work	I34
Manufacture of Furniture	I15		Manufacture of Artwork and Other Manufacturing	I35
Manufacture of Paper and Paper Products	I16		Recycling and Disposal of Water	I36
Printing, Reproduction of Recording Media	I17		Production and Distribution of Electric Power and Hear Power	I37
Manufacture of Articles for Culture, Education and Sport Activity	I18		Production and Distribution of Gas	I38
Processing of Petroleum, Coking, Processing of Nuclear Fuel	I19		Production and Distribution of Water	I39
Manufacture of Raw Chemical Materials and Chemical Products	I20		Other Sectors	I40
